# The Teramoto distal tibial oblique osteotomy (DTOO): surgical technique and applicability for ankle osteoarthritis with varus deformity

**DOI:** 10.1007/s11751-018-0307-0

**Published:** 2018-01-29

**Authors:** Tsukasa Teramoto, Shota Harada, Motoyuki Takaki, Tomohiko Asahara, Narutaka Kato, Nobuyuki Takenaka, Takasi Matsushita, Yosiaki Makino, Kouitiro Tasiro, Ootuka Kazutaka, Yukinobu Nishi, Kiyoto Kinugsa

**Affiliations:** 1 Department of Traumatology, Trauma and Reconstruction Centre, Southern Tohoku General Hospital, Fukushima Medical University, 7-115 Yatsuyamada Koriyama, Fukushima, Japan; 2 Nagasaki-ASAMI, Nagasaki, Japan; 3 Department of Orthopaedic Surgery, Chikamori Hospital, Kōchi, Japan

**Keywords:** Ankle, Tibia, Deformity, Distal tibial oblique osteotomy, Varus, Ankle osteoarthritis

## Abstract

We have devised a medial peri-articular osteotomy, the distal tibial oblique osteotomy (DTOO), and have used this technique since 1994 for ankle osteoarthritis of advanced and late stages associated with varus inclination. This report describes the surgical technique and its applicability. DTOO can be used for cases of varus ankle osteoarthritis with a range of the ankle joint movement of at least 10° or more. The osteotomy is obliquely directed cut across the distal tibia from proximal-medial to distal lateral and is of an opening-wedge type with the centre of rotation coincident with the centre of the tibiofibular joint. A laminar spreader instrument is inserted in the osteotomy to open the wedge until the lateral surface of the talar body is seen on X-ray to be in contact and congruent with medial articular surface of the lateral malleolus. Common obstacles which may prevent this contact and congruency are bony spurs present on the anterior side of fibula or on the lateral side of the tibia; these require removal. The opening-wedge osteotomy is held in position by an Ilizarov external fixator or internally fixed with a plate. Bone graft is taken from the iliac crest and inserted into the open wedge. If, after completion of the osteotomy, the dorsiflexion angle of the ankle joint does not exceed 0°, a Z-lengthening is performed of the Achilles tendon. In the DTOO for ankle osteoarthritis, the contact area of the ankle joint increases and decreases the load pressure per unit area. Furthermore, as the width of the ankle mortice is restored through the realignment of the body of the talus, instability at the ankle joint decreases. There is additional improvement with restoration of the inclination of the distal tibial articular surface as this directs the hindfoot valgus and corrects the alignment of the foot, with consequent improvement of ankle pain.

## Introduction

Historically, supramalleolar low tibial osteotomies (LTO) [12–14], total ankle arthroplasty (TAA) [6, 7] and arthrodesis of the ankle (AA) [1–3] have been performed for the surgical treatment for ankle osteoarthritis. It is accepted generally that the LTO is not suited to advanced or late stages of osteoarthritis [13, 14]. Although it is considered that the TAA maintains a range of the ankle joint movement, it not suited for patients who anticipate manual work or active leisure pursuits as daily undertakings [6, 7]. Ankle arthrodesis (AA) is used for cases of advanced and late stages of osteoarthritis whilst it is successful in producing a stable and pain-free ankle, it sacrifices mobility, and there is the possibility of degenerative changes in adjacent joints with time.

We have devised a peri-articular osteotomy, the distal tibial oblique osteotomy (DTOO) and have used this technique since 1994 for varus ankle osteoarthritis of advanced and late stages. This achieves the objective of maintaining mobility of the ankle joint and reconstructing the ankle mortise such that it is capable of high demand activity and is accompanied with good clinical results. DTOO is a surgical technique able to realign the ankle joint surface without cutting the fibula. The purpose of this report is to describe the surgical technique and its applicability for varus ankle osteoarthritis.

## Clinical indications for the procedure

Takakura classified varus ankle osteoarthritis into four stages using weight-bearing radiographs: [12].

*Stage 1* No joint space narrowing but early sclerosis and osteophyte formation.

*Stage 2* Narrowing of the joint space medially.

*Stage 3* Obliteration the joint space with subchondral bone contact medially.

*Stage 4* Obliteration of the whole joint space with complete bone contact.

In clinical use, a simplified form of the classification describes stage 1 as early, stage 2 and 3 as intermediate, and stage 4 as late. Tanaka further classified stage 3 into stages 3a and 3b. In stage 3a, the obliteration of the joint space was limited to the medial malleolus and in stage 3b, the obliteration extended to the roof of the dome of the talus. Tanaka concluded that LTO can be used for up to stage 3a according to this classification for ankle osteoarthritis, and that it is not indicated for advanced nor the late stages 3b and 4 [13, 14]. In comparison, the DTOO is indicated for all stages of varus ankle osteoarthritis as long as there is a movable range within the joint, typically at least 10° or more [9, 10]. Use of this technique should be based on taking into consideration the patient’s social, work and sports activities—regardless of patient’s age or stage of ankle osteoarthritis as based on the classification by Tanaka. However, the DTOO is contraindicated in patients with joint degeneration after infected arthritis, rheumatoid arthritis, a Charcot joint and steroid-induced arthropathy.

## Surgical technique

### Osteotomy stabilisation with an Ilizarov external fixator

**Fig. 1:**
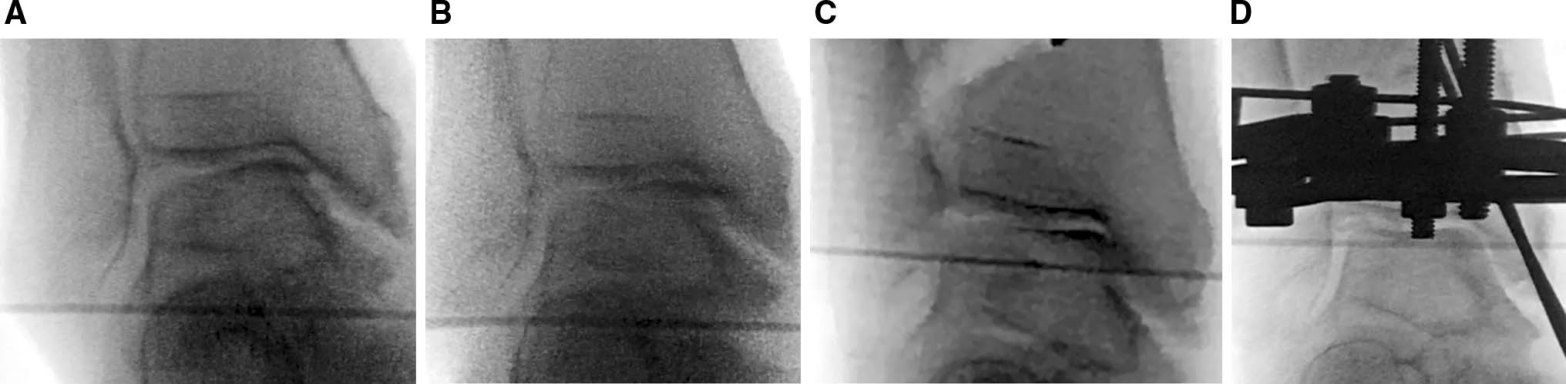
Varus and valgus stressing of the ankle is performed and illustrates instability from the tilt of the talar body within the ankle mortise and the widening of the lateral gutter before the DTOO (**a**, **b)**. After DTOO, the lateral gutter is narrowed from contact between the articular surface of the lateral aspect of the talar body and the medial articular surface of the lateral malleolus (**c)**. Fixation is with Ilizarov external fixation and supplementary Kirschner wires through the medial malleolus (**d**)

The operation is performed with general anaesthesia and in the supine position. Varus and valgus stress views of the ankle are performed to confirm the instability of the ankle under anaesthesia (Fig. 1a, b).

A skin incision 3 cm long is made about 7 cm proximal to the tibial articular surface on the medial side and a subperiosteal exposure created. Using X-ray image intensification, a check on the starting point of the osteotomy is made to ensure it is about 6 cm proximal to the tibial articular surface. The cut is made obliquely towards the centre of the tibiofibular joint. A 2-mm Kirschner wire is driven from the proximal-medial to distal lateral in the coronal plane and towards the centre of the tibiofibular joint; this guide can be used to direct the osteotome for the osteotomy.

**Fig. 2:**
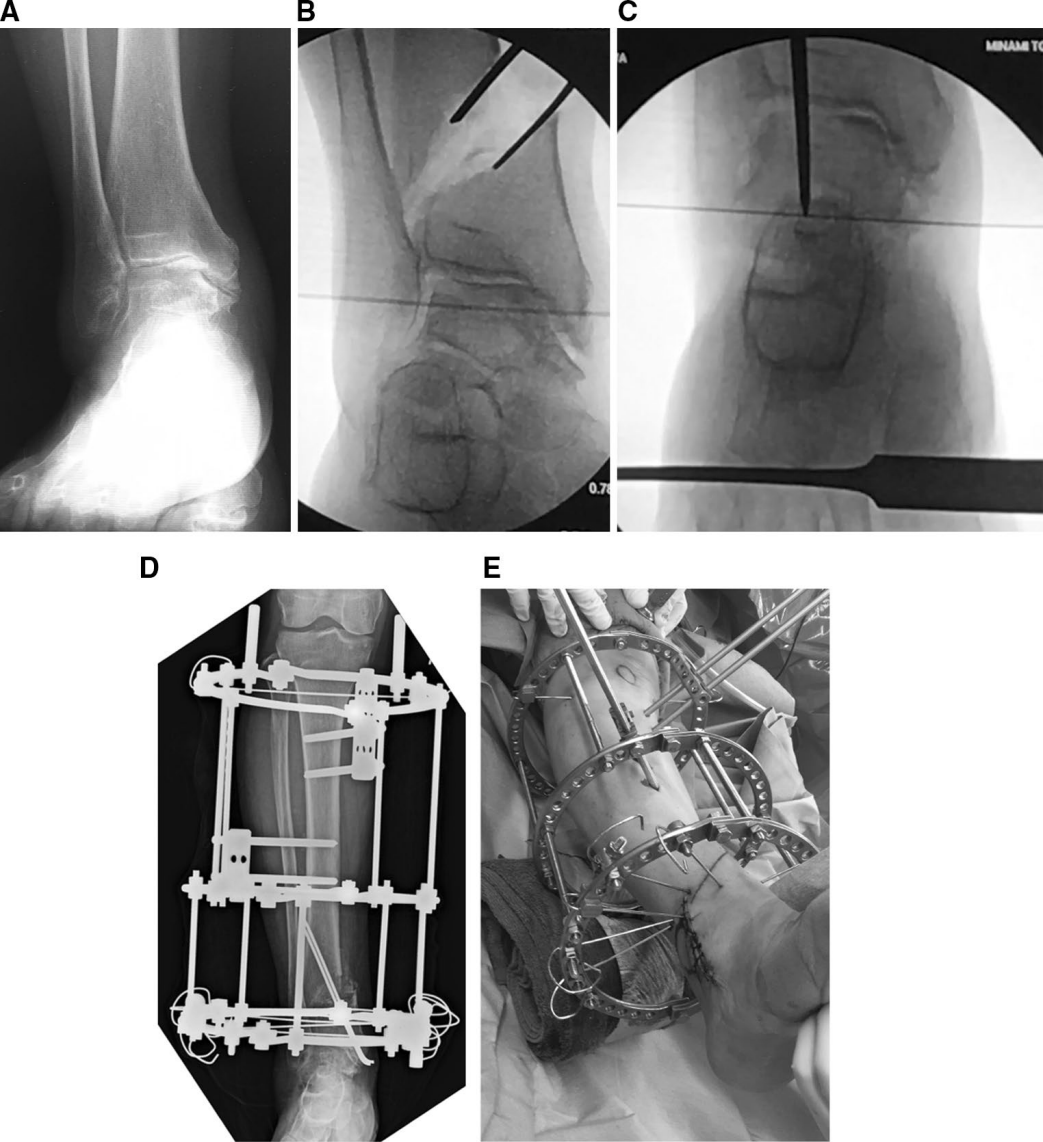
A 61-year-old woman with stage 3b of ankle osteoarthritis in her right ankle was treated with a DTOO. The weight-bearing radiograph before DTOO showed the joint space narrowing and the obliteration extended to the roof of the dome of the talus (**a**). The second radiograph shows separation of the osteotomy by laminar spreader until the lateral talar articular surface comes into contact with the distal medial fibular articular surface (**b**). Bony spurs present in the fibula and tibia are removed because of impingement during the corrective osteotomy. The final radiograph shows that the plantar surface of the foot becomes perpendicular to the tibial axis after osteotomy (shown by the overlying osteotome to indicate the tibial axis) (**c**). Fixation is with the Ilizarov external fixator (**d**, **e**)

Once the osteotomy is complete, a laminar spreader is inserted in order to create an open wedge gradually. The separation of the surfaces of the osteotomy is increased with x-ray guidance until the articular surface of the lateral part of the talar body comes into contact with the articular surface of the medial part of the distal fibula (Fig. 2b). In some instances, the point at which the open wedge is created is not exactly in the mid-sagittal axis but slightly anterior or posterior, indicating this correction may occur not just in the coronal plane but in an oblique plane. For some cases, rotation can also be included. This suggests the osteotomy can be a correction in three dimensions and not just in the coronal plane. Obstacles which may prevent this restoration of alignment and coaptation of articular surfaces include the presence of bony spurs on the anterior side of the fibula or the lateral side of the tibia; if identified, these should be removed. After widening of the osteotomy, the plantar surface of the foot becomes perpendicular to the tibial anatomical axis. Varus and valgus stress assessments of the ankle should be performed to confirm that the instability of the ankle has been improved.

Ilizarov rings are applied in three locations: just below the proximal tibial tubercle (A), tibial diaphyseal region (B) and just above the ankle (C). The first Ilizarov ring is fixed in place perpendicularly to the tibial shaft just below the tibial tubercle using two wires and two 6 mm half pins. The clearance between the front of the tibia and the ring is set to approximately three finger breadths (Fig. 2d, e). Next, the Ilizarov ring in the diaphyseal region is fixed in place with two 6-mm half pins. Finally, the distal bone fragment is fixed in place with five Ilizarov wires. On this last ring, the most distal Ilizarov wire is inserted parallel to the middle Ilizarov ring (B—diaphyseal region). This Ilizarov ring is positioned just above the ankle joint (C) to be parallel to the Ilizarov ring (B) of the diaphysis. Next, more Ilizarov wires are inserted posterior to the fibula forwards in a medial direction; additional wires are inserted in safe corridors around the ankle such that the distal ring(C) is secured by 4 or 5 Ilizarov wires. After confirming that the bone fragment is fixed in place and does not move after removing the spreader, bone graft is taken from the iliac crest and transplanted. Recently, two 2.4-mm Kirschner are added percutaneously from the medial malleolus to augment stability and removed once healing is complete (Fig. 2d).

In those patients in whom the dorsiflexion angle of the ankle joint does not exceed plantigrade after completion of the osteotomy and bone grafting, a Z-lengthening is performed on the Achilles tendon. Finally, the wound is lavaged and a Penrose drain is placed prior to closing the wound. After the operation, attention should be paid for potential skin necrosis; in many cases, the medial skin gets tense after the correction with possibility of necrosis or blistering.

The Ilizarov external fixator may not be suited for some clinical cases. When a hemiarthroplasty or total knee arthroplasty has been used for the ipsilateral knee joint or if the Ilizarov external fixator is declined by the patient, locking plate fixation is used. For such cases, the tension created on the medial side makes plate insertion on the same medial surface difficult, and as such, a compromise to the degree of correction has to be made in order to facilitate skin closure over the plate. This is a strong argument for using the Ilizarov external fixator for fixation of the osteotomy [9–11]. Alternatively, the problem of tension on the medial skin can be reduced if some bone is removed from the cortex on the medial side of the distal tibia to facilitate adequate correction and wound closure without tension. We have noted that if there is no previous scar on the medial skin and the osteotomy separation is less than 15 mm, a locking plate can be inserted without removing the medial tibial cortex and cancellous bone. In any case, the most important point is to avoid an insufficient correction just to facilitate plate insertion and wound closure [9, 10].

### Osteotomy stabilisation with a locking plate

**Fig. 3:**
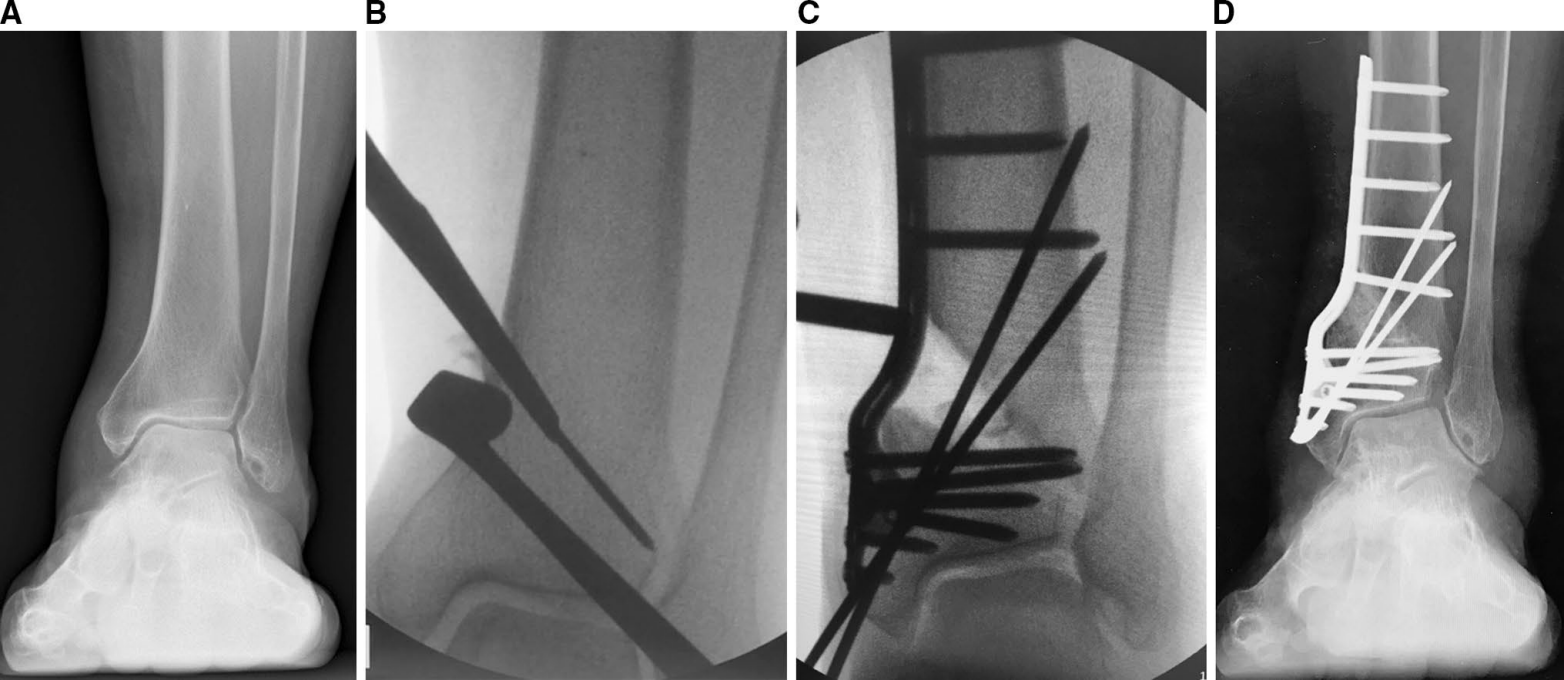
A 76-year-old female with stage 2 varus ankle osteoarthritis of her left ankle. She had bilateral total knee replacements. Weight-bearing radiographs before DTOO showed the narrowing of the joint space medially (**a**). The intra-operative radiograph shows the DTOO performed with a chisel (**b**). After separation of the osteotomy surfaces to the optimum degree, fixation was established with a curved plate (**c**). As ankle dorsiflexion after the osteotomy was −15°, the Achilles tendon was elongated (30 mm). The weight-bearing radiograph 1 year after DTOO shows the good preservation of ankle joint space with the sole of the hindfoot perpendicular to the axis of the tibia (**d**). The moderate instability of ankle joint present before DTOO has been improved and ankle joint pain resolved completely such that the patient could run. The range of motion was maintained after DTOO (dorsiflexion 5°—plantar flexion 20°)

A 14-cm vertical incision is made over the medial distal tibia. The procedural details for anaesthesia, patient positioning, osteotomy, separation of the osteotomy and confirmation of the restoration of ankle joint stability are the same as when using the Ilizarov external fixator. After separation of the osteotomy by the laminar spreader to a sufficient degree, the medial tibial cortex bone and cancellous bone on which the locking plate is to be applied is removed to allow the plate to be fixed and wound closure achieved without undue skin tension. The removed cortex and cancellous bone are used for bone graft and, if insufficient, supplemented with additional bone graft from the iliac crest (Fig. 3a–d).

### Post-operative therapy

**Fig. 4:**
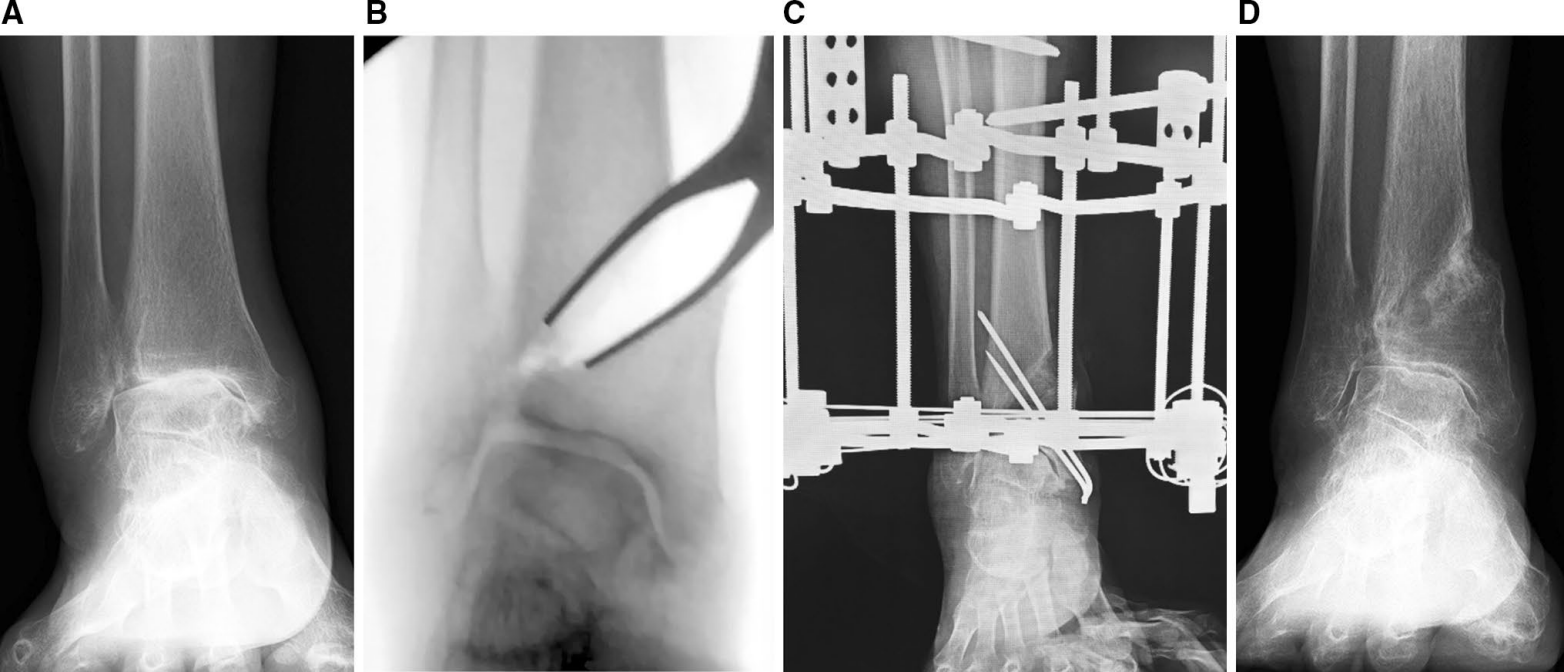
A 71-year-old woman with stage 3b varus ankle osteoarthritis of her right ankle underwent a DTOO. The weight-bearing radiograph before DTOO showed joint space narrowing and even bone-to-bone contact over the dome of the talus (**a**). The intra-operative radiograph shows separation of the DTOO by laminar spreader (**b**). The separation was continued until the articular surface of the lateral talar body came into contact with the medial articular surface of the lateral malleolus. Osteotomy fixation was with the Ilizarov external fixator and supplementary Kirschner wires (**c**). Weight-bearing radiographs 2 years after DTOO shows good restoration of ankle joint and with the plantar surface of the hindfoot perpendicular to the tibial axis (**d**). Clinical review confirmed complete resolution of ankle joint pain. The range of motion was decreased slightly after DTOO (dorsiflexion 0°—plantar flexion 15°), but the patient was able to return to farm work

**Fig. 5:**
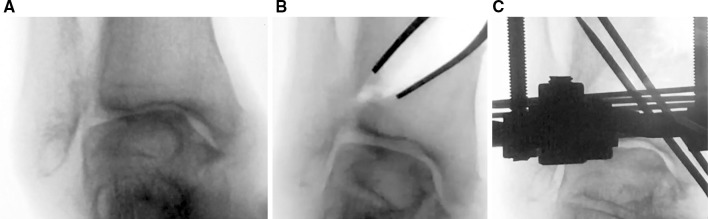
A varus and valgus stress of the ankle is performed to confirm the instability (**a**, **b**). The same was repeated after DTOO (**c**, **d**). The instability of ankle has improved. Fixation is with the Ilizarov external fixator and supplementary Kirschner wires (**c**)

**Fig. 6:**
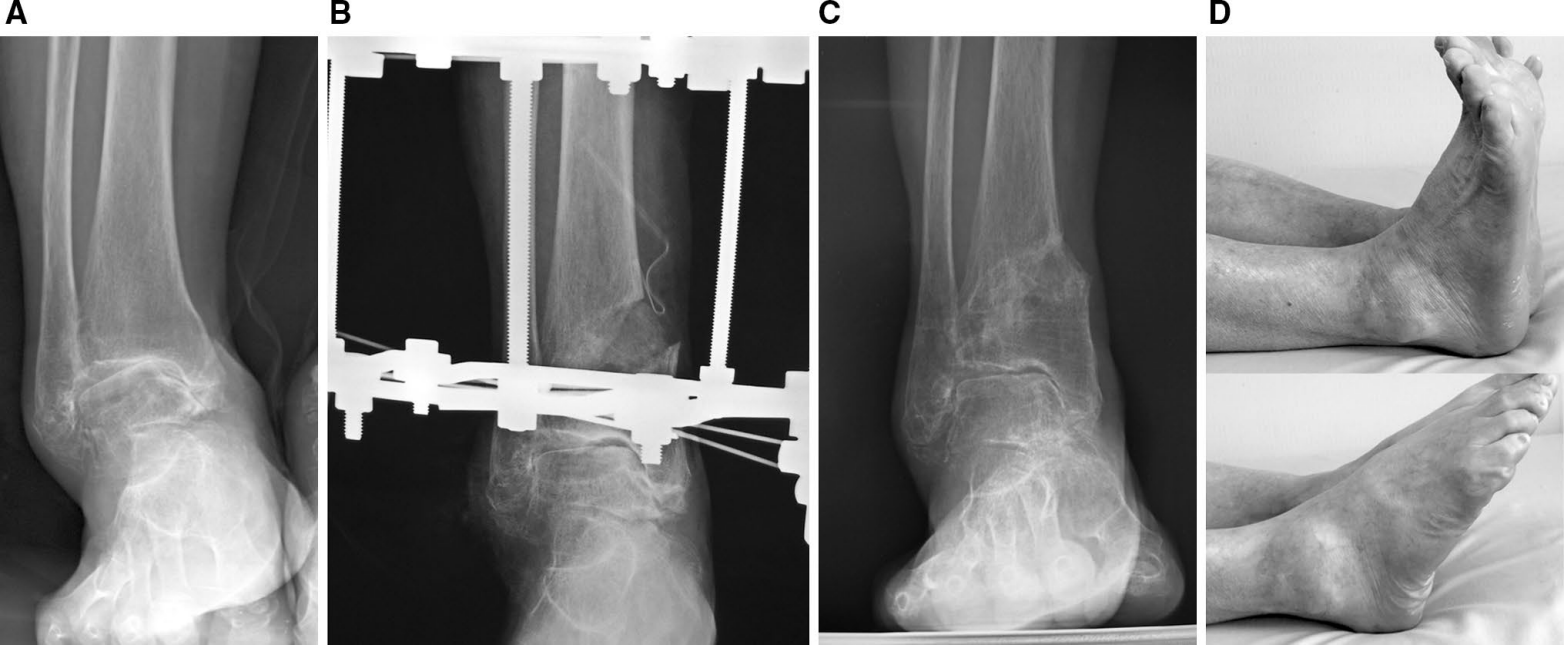
A 69-year-old woman with stage 3b varus osteoarthritis in her right ankle underwent a DTOO. The weight-bearing radiograph before surgery showed the severe varus deformity and joint space narrowing with obliteration of this joint space on the roof of the dome of the talus (**a**). During surgery, the dorsiflexion measured -10° after osteotomy; the Achilles tendon was elongated to overcome this equinus. Bony spurs present in the fibula and the tibia were removed owing to the impingement caused. The tibia and osteotomy was fixed by an Ilizarov external fixator (**b**). The instability of ankle joint before DTOO was improved with the talar articular surface and plantar surface of the hindfoot almost perpendicular to the tibial axis (**b**, **c**). The severe ankle joint pain was resolved completely. The range of motion was maintained 2 years after DTOO (dorsiflexion 0°; plantar flexion 30°) (**d**). The patient could return to farm work without pain

**Fig. 7:**
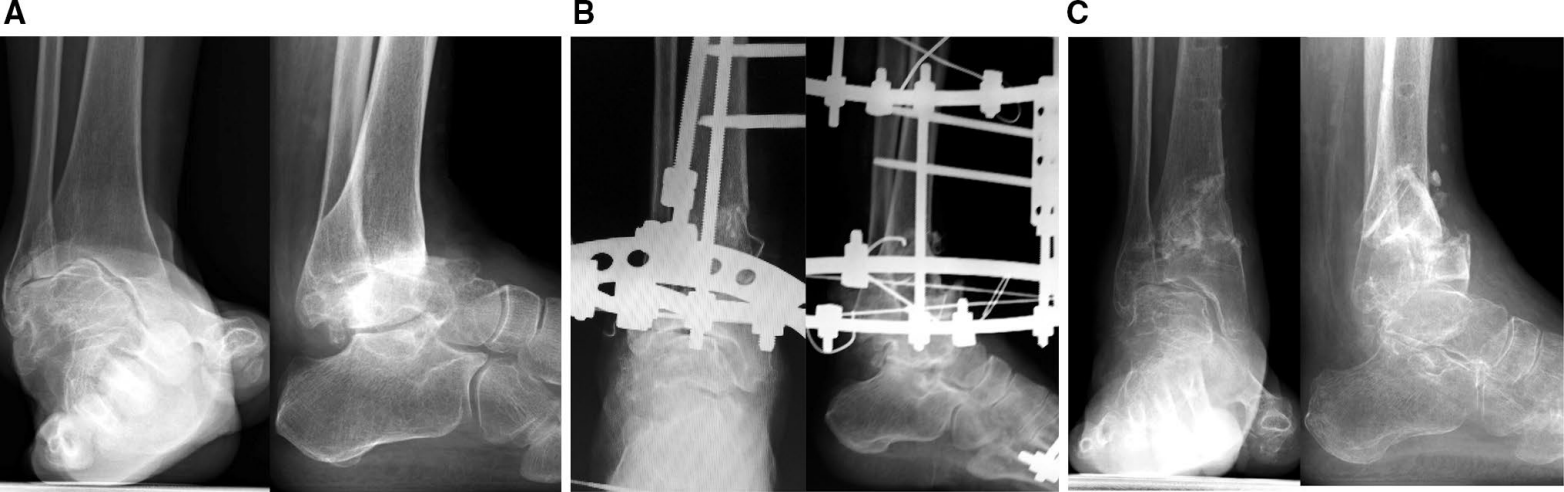
A 70-year-old woman with stage 4 varus ankle osteoarthritis of her right ankle underwent a DTOO. The weight-bearing radiograph before surgery shows severe varus deformity and joint space narrowing (**a**). During surgery and after the osteotomy, ankle dorsiflexion was reduced to − 10°; the Achilles tendon was elongated (20 mm). Bony spurs present in the fibula and the tibia are removed because these spurs prevented satisfactory restoration of ankle joint congruity. The severe ankle joint instability and subluxation was improved after the DTOO. Fixation was by Ilizarov external fixator (**b**). Weight-bearing radiographs 2 years after the DTOO show the good restoration of the ankle mortise, with the plantar surface of the hindfoot perpendicular to the tibial axis (**c**). The range of motion was maintained (dorsiflexion 5°—plantar flexion 25°). The patient reported absence of symptoms and a return to activities of daily living without pain

Ankle joint movement is encouraged post-surgery and partial weight bearing allowed through the external fixator at 6–8 weeks. The Ilizarov external fixator is removed 3 months after surgery (Figs. 4a–d, 5a–c, 6a–d, 7a–c).

## Discussion

The surgical strategies of intra-articular debridement (IAD) [4, 8], low tibial osteotomies (LTO) [12–14], total ankle arthroplasty (TAA) [6, 7], arthrodesis of the ankle (AA) [1–3] and distraction arthroplasty (DA) [5] can be used for treatment of ankle osteoarthritis. IAD is suitable for early-stage ankle osteoarthritis but not for advanced nor late stages [4, 8]. In comparison, LTO is indicated for up to stage 3a (Tanaka classification for ankle osteoarthritis) but not for advanced or late stages 3b and 4 [13, 14]. TAA maintains ankle joint movement and is suited for advanced and late stages; it is not suited for patients where there is severe deformity associated with the distal tibial articular surface, and furthermore, sports and heavy manual work activities are limited after surgery [6, 7]. In contrast, AA [1–3] is a surgical technique for advanced and late stages of ankle osteoarthritis as it is possible to have high levels of pain-free function, but there is loss of ankle joint movement and a risk of arthrosis of adjacent joints in the future. Distraction arthroplasty is a method with a possibility of the joint cartilage repair but current results are mixed with reports of patients with better pain scores and some without any improvement [5].

In DTOO, the shape of the ankle joint surface is changed by cutting and tilting the tibial plafond without osteotomy of the fibula. The inclination of the distal tibial articular surface with respect to the tibial axis is altered and with associated improvement in ankle stability [9, 10]. The oblique osteotomy is dilated with a spreader until the lateral talar articular surface comes into contact with the medial articular surface of the lateral fibular malleolus; this is achieved whilst contact is maintained between the medial malleolar articular surface and the medial talar articular surface. Therefore, in DTOO, the ankle mortise congruency is restored such that medial and lateral articular gutters on either side of the talar body are symmetrical. This implies that the contact area of the talar articular surface is increased with the tibia and fibula by performing the DTOO; with the contact area increased, load pressure is dispersed across the ankle joint, and there is a decrease in the load pressure per unit area applied to the ankle joint. This degree of improved congruency between the articular surfaces of the talar dome and distal tibial plafond and that in the medial and lateral gutters of the ankle mortise improves ankle stability [9, 10].

## Conclusion

The distal tibial oblique osteotomy (DTOO) is a procedure suited for the ankle osteoarthritis by improving the contact area of the ankle joint and decreasing the load pressure per unit area. This is accomplished with an improvement in ankle stability and a restoration of the hindfoot valgus. Clinical results confirm a reduction in ankle pain and an ability to perform hard manual work or return to sports activities.
